# Symptoms of Cancer of the Uterus

**Published:** 1907-03

**Authors:** 


### SYMPTOMS OF CANCER OF THE UTERUS.

J. G. Clark says:

The evils of procrastination in the diagnosis of uterine cancer are emphasized in this article, and the old notion that marked irregularities, floodings and leucorrhea at the menopause are normal in any sense should be done away with. Another fallacy with serious consequences is the hereditary theory, the assumption too common in the past, that symptoms could not mean cancer because there was none in the family history. The fact that cancer is a disease of middle life, also cannot be too strongly emphasized. As shown by a chart, at least 90 per cent. of the cases occur between the ages of 40 and 50. Another fact on which stress has been laid is the importance of injuries of childbirth as a causative factor. Sampson has shown that in 412 cases only 3 per cent. had not been pregnant. The suggestive signs calling for an exhaustive examination in a woman between 22 years of age and the menopause are given by Clark as follows: "1. Any deviation of the menstrual period in the way of an excess or an inter-menstrual discharge, especially in women beyond 30 years of age. The most suspicious of these are: (a) a mere show after slight exertion, defecation or coitus; (b) increasing length of the period, even if only one day more than has been her established habit. Every woman is a law to herself in this respect. 2. An exacerbation in amount or change in character of the discharge in a woman who may have had a simple leucorrhea for months or years. Of these changes a free aqueous, acrid, or blood-tinged discharge is especially portentous. 3. A leucorrheal discarge in a patient who has never had it before. 4. Every atypical discharge in a woman after the menopause. These individuals are especially liable to cancer and should, if possible, be even more exhaustively examined. 5. Pelvic pain of more than a few days' duration should be an urgent reason for examination, although it is very seldom an early symptom of cancer." Clark insists on the use of the microscope, especially when in doubt as to the diagnosis from the early symptoms. In case of cancer of the fundus of the uterus the microscope becomes the principal diagnostic aid.—Jour. A. M. A., December 8, 1906.

